# Patients’ Experiences of Cancer Diagnosis as a Result of an Emergency Presentation: A Qualitative Study

**DOI:** 10.1371/journal.pone.0135027

**Published:** 2015-08-07

**Authors:** Georgia Black, Jessica Sheringham, Vicki Spencer-Hughes, Melanie Ridge, Mairead Lyons, Charlotte Williams, Naomi Fulop, Kathy Pritchard-Jones

**Affiliations:** 1 UCL Department of Applied Health Research, UCL, London, England; 2 UCL Partners, London, England; 3 LKSS Public Health Training Programme & Public Health Service, Lambeth and Southwark Local Authorities, London, England; Centre for Health and Population Sciences, Hull York Medical School, UNITED KINGDOM

## Abstract

**Introduction:**

Cancers diagnosed following visits to emergency departments (ED) or emergency admissions (emergency presentations) are associated with poor survival and may result from preventable diagnostic delay. To improve outcomes for these patients, a better understanding is needed about how emergency presentations arise. This study sought to capture patients' experiences of this diagnostic pathway in the English NHS.

**Methods:**

Eligible patients were identified in a service evaluation of emergency presentations and invited to participate. Interviews, using an open-ended biographical structure, captured participants' experiences of healthcare services before diagnosis and were analysed thematically, informed by the Walter model of Pathways to Treatment and NICE guidance in an iterative process.

**Results:**

Twenty-seven interviews were conducted. Three typologies were identified: A: Rapid investigation and diagnosis, and B: Repeated cycles of healthcare seeking and appraisal without resolution, with two variants where B1 appears consistent with guidance and B2 has evidence that management was not consistent with guidance. Most patients’ (23/27) experiences fitted types B1 and B2. Potentially avoidable breakdowns in diagnostic pathways caused delays when patients were conflicted by escalating symptoms and a benign diagnosis given earlier by doctors. ED was sometimes used as a conduit to rapid testing by primary care clinicians, although this pathway was not always successful.

**Conclusions:**

This study draws on patients' experiences of their diagnosis to provide novel insights into how emergency presentations arise. Through these typologies, we show that the typical experience of patients diagnosed through an emergency presentation diverges significantly from normative pathways even when there is no evidence of serious service failures. Consultations were not a conduit to diagnosis when they inhibited patients’ capacity to appraise their own symptoms appropriately and when they resulted in a reluctance to seek further healthcare.

**Recommendations:**

The findings also point to potentially avoidable breakdowns in the diagnostic process. In particular, to encourage patients to return to the GP if symptoms escalate, a stronger emphasis is needed on diagnostic uncertainty in discussions between patients and doctors in both primary and secondary care. To improve appropriate access to rapid investigations, systems are needed for primary care to communicate directly with secondary care at the time of referral.

## Introduction

England still lags behind many comparable European nations and others globally in one-year survival for common cancers [1]. Delays in diagnosis have been proposed as one reason for this poorer survival. One particular cause for concern is that 25% of patients are diagnosed through an emergency presentation, i.e. after visiting an Emergency Department (ED, also commonly referred to as A&E in England) or an emergency admission to hospital [2]. Short-term survival in these patients is poor compared with other routes to diagnosis even when age and case mix are taken into account [3]. Emergency presentations are monitored at local levels in England as a possible indicator of preventable diagnostic delay [4].

There is evidence that emergency presentations are socially patterned, with older patients consistently more likely to be diagnosed as emergencies than younger patients [2,5,6]. Raine *et al* (2010) found the risk of emergency presentations for lung and colorectal cancer was highest amongst men and in patients living in the most deprived areas. However, a study in North East London [5] did not find any variation by gender or deprivation amongst colorectal cancer patients.

It is still not well understood how emergency presentations arise or to what extent they are preventable. Hamilton [7] cautions that emergency presentations may not always be due to preventable failure in the diagnostic process and Rubin *et al* [8] observe that a range of factors may contribute across the entire cancer pathway. Qualitative studies of cancer patients provide some insight into why longer diagnostic intervals may occur due to delays in patient presentation [7,8]. For example, patients may defer seeking care when they have intermittent symptoms or are unaware of the implications of specific symptoms [9,10]. This could lead to emergency presentations if patients only seek help when symptoms are at crisis point. Evidence suggests most patients diagnosed as emergencies seek primary care before their diagnosis [5], but factors affecting the diagnostic process after this initial help-seeking are less well described. Poor outcomes may arise for patients diagnosed following emergency presentation for reasons other than diagnostic delay too. For example, King *et al* (2011) found an absence of a clear pathway from the emergency department, which for patients diagnosed following an ED visit, was a cause of poor patient experience [11].

A detailed understanding of what leads to an emergency presentation is therefore needed. In this study, we sought to capture in detail the experiences of patients whose cancer was diagnosed following an ED visit to understand how emergency presentations arise and identify where there is scope to improve outcomes. We have focussed specifically on service and pathway issues, rather than participants’ appraisal of specific body symptoms as this is covered comprehensively in cancer literature[12–14].

## Materials and Methods

### Setting

This study was nested within a service evaluation led by *London Cancer*, an integrated cancer system covering the 3.5 million population of North Central and North East London, and West Essex. This evaluation identified 963 patients between Dec 2012 and Aug 2013 with cancer diagnosed after presentation in eleven EDs in nine acute NHS Trusts. Recruitment was open to all nine trusts, although we only received participants from seven.

### Recruitment

We sampled purposively to obtain a range of cancer tumours and demographic characteristics. A member of the clinical team gave patients that completed a questionnaire as part of the overall service evaluation (n = 104) an information sheet about the interview study. Patients were eligible if they were over 18 years old, newly diagnosed with any cancer following an emergency presentation, with capacity to understand the consent procedures. The clinical team did not invite patients they considered too ill to support the consent and interview procedures to participate.

Researchers telephoned people that expressed interest in participation one week later to explain the study and arrange an interview date. Before the interview commenced, written consent was obtained.

### Data collection

Interviews were conducted (April-October 2013) by GB, MR and VSH and two other researchers in hospital or in participants’ homes. Three interviews were conducted with a spouse or relative present, at participants’ request. A topic guide developed with cancer patient representatives was adapted to an open-ended biographical structure (“*how did this all start for you*?”) to better capture the complexity of patients’ interactions with healthcare services before diagnosis. Interviews were audio-recorded and transcribed via a confidential service, which removed identifying information.

### Analysis

**Fig 1:**
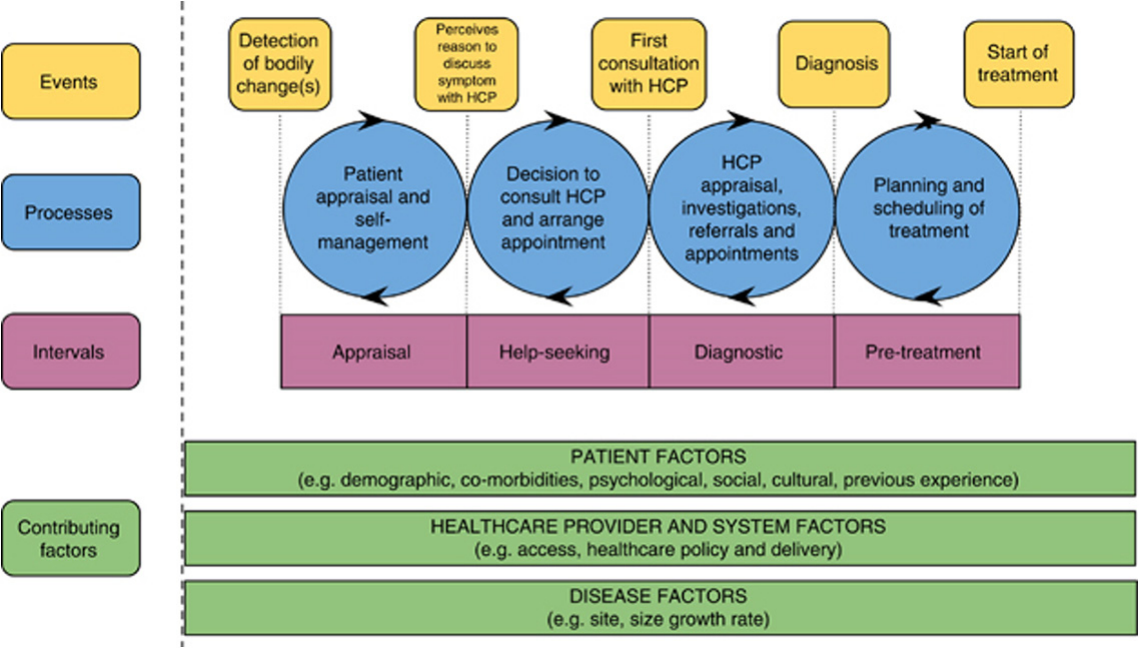
Walter's Model of Pathways to Treatment (from Walter et al., 2011[15]).

The framework of Walter’s model of Pathways to Treatment was used as an organising and interrogating construct to produce findings comparable to other literature on early diagnosis in cancer [14,15]. This model considers the contribution of patients, providers/system and disease factors to four intervals: (Symptom) Appraisal, Help-seeking, Diagnostic and Pre-treatment (Fig 1). It is presented mainly as a linear sequence leading to diagnosis but acknowledges the possibility that patients move back and forth between intervals in consultation with healthcare professionals (HCPs).

The model was used at a number of points in the analysis process: first, all the data from the interviews were coded using *NVivo* software [16] into the four ‘Processes’ from the model plus an extra code specifically related to Diagnosis as they applied directly to the participant.

Data that did not fit into these categories were coded inductively. Coded data were reviewed and explored. In selected cases, pictorial constructions of participants’ pathways were constructed from interview data to summarise the approximate sequence and duration of all healthcare activity recalled by participants.

We compared the coded data on similar parts of the pathway between the participants. This process of comparison and discussion generated ideas about the participants’ experiences that were refined into themes using supporting quotations. We used the intervals of Walter’s model to create participant pathway diagrams to exemplify case studies. We compared these individual experiences with the themes we had generated to try to elucidate *why* and *how* emergency medical services were used as part of the cancer pathway.

Finally, we examined patients by demographic characteristics known to be associated with emergency diagnosis (gender and age). We next considered whether their cancer site was commonly considered detectable at an early stage through screening or symptom recognition (lung, ovarian, colorectal) or not (head & neck, haematological, upper GI).

Building on this initial coding, we developed three typologies informed by the NICE referral guidelines for suspected cancer as a benchmark for categorising GP behaviour [17]. These methods are consistent with a deductive-inductive hybrid thematic analysis approach [18].

### Ethics

The study was approved by The National Research Ethics Service Committee for South East Coast—Kent (Ref: 12/LO/1477). NHS research governance was obtained at all trusts.

## Results

### Sample

The sample comprised 27 people from seven trusts. Fifty individuals were recruited, but 20 were not interviewed when they became too unwell to participate, died, or changed their mind. Three patients could not take part because it was not possible to schedule an interview at a convenient time within the study duration.

Seven cancer types were recorded: colorectal, upper GI, lung, haematological, head and neck, gynaecological, and unknown primary; the most common diagnosis was colorectal cancer (41%). Ages ranged from 18 to 92 years (Table 1).

**Table 1:** Participant characteristics.

Characteristic	n (%)
**Cancer site**	
**Colorectal**	11 (41%)
**Lung**	4 (15%)
**Head and Neck**	4 (15%)
**Haematological**	3 (11%)
**Other^a^ (CUP, Upper GI, Ovarian)**	5 (15%)
**Gender: Male**	15/27 (56%)
**Ethnicity^b^: White British**	17/23 (74%)
**Age: Median (range)**	59 (18–92)

^a^ CUP = cancer of unknown primary site; Upper GI = upper gastrointestinal, e.g. oesophageal, pancreatic

^b^ Ethnicity data missing in 4 cases. Participants that did not identify as White British identified as White other, Mixed, Black Caribbean.

Participants’ accounts fitted at least one of the following typologies:

A: Rapid investigation and diagnosis after detection of bodily changes
B: Repeated cycles of help-seeking and appraisal with two variants:
  B1) Management appears consistent with NICE guidance
  B2) Evidence that management was inconsistent with NICE guidance or other evidence of service failure

There was a clear distinction between typology A and B but three patient experiences within typology B have aspects of both B1 and B2, hence considering this as one typology with two variants.

**Table 2:** Participant typologies with diagnostic and demographic information.

Typology	Constituent participants with diagnostic & demographic information*			
		Older ages		Younger ages
A:	1	Lung, M	11	Colorectal, M
	7	Upper GI, M		
	9	Haem, M		
B1:	2	Colorectal, F	3	Lung, M
	17	Head & Neck, M	4**	Colorectal, M
	18	Head & Neck, M	5	Colorectal, F
	21	Unknown primary, F	16	Haem, M
	22	Lung, M	19	Haem, F
	23**	Colorectal, M	27	Colorectal, M
	24**	Colorectal, F		
	25	Colorectal, M		
	26	Colorectal, F		
B2:	6	Head & Neck, F	4**	Colorectal, M
	10	Lung, M	8	Gynae, F
	12	Gynae, F	15	Colorectal, F
	13	Unknown primary, F	20	Upper GI, F
	14	Colorectal, M		
	23**	Colorectal, M		
	24**	Colorectal, F		

*M = Male, F = Female.

**Participants’ experiences fitted both typologies B1 and B2

### Description of typologies

As shown in Table 2 there were no clear patterns in the distribution of patients by age, gender or cancer type between typologies. In addition, we found no consistent pattern between age, gender, and the types of symptoms experienced, or their interpretation or appraisal by the participant. Participants often presented their ED visit as an inevitable consequence of the appearance of alarming symptoms. However, other participants experiencing similar symptoms chose primary care. For example, acute abdominal pains and constipation in three colorectal cancer patients resulted in two patients calling an ambulance, and a third visiting the GP.

We now describe the nature of the typologies generated with examples and quotations to support them.

#### Typology A: Rapid investigation and diagnosis after detection of bodily changes

This typology is defined by a short, normative pathway with respect to the Walter model [15]: participants identify a problem, decide to seek help, are seen by a healthcare practitioner and receive a diagnosis in a timely fashion (N = 4). Two participants were taken by ambulance, and two were sent by their GP. A common feature of this typology was the sudden onset of dramatic or visible symptoms such as confusion, a swollen leg, jaundice and loss of consciousness.

**Fig 2:**
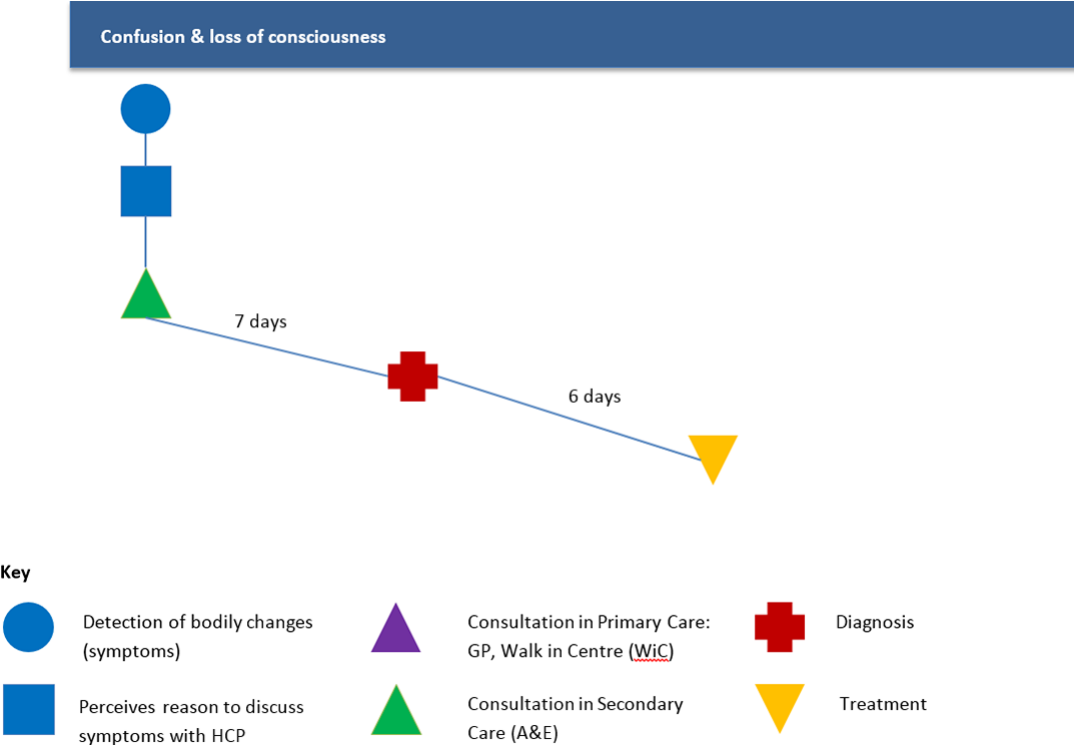
Pathway to treatment illustrating typology A: Participant 1.

We present a case study of Participant 1 (Fig 2). Following the Walter’s model, this man felt very tired in the afternoon (patient symptom appraisal) and went to bed (self-management). He awoke in a confused state *(“I found that I was in the shower fully clothed” Participant 1*, *M*, *Lung*) and rang a colleague. The colleague rang his daughter and together they called for an ambulance (decision to consult HCP). In the ED the participant had several scans and was admitted (HCP appraisals and investigations). Investigations over the next three days confirmed a cancer diagnosis.

#### Typology B: Repeated help-seeking and appraisal

In contrast to participants in typology A, participants described repeated cycles of help-seeking and appraisal before their eventual diagnosis. Participants detected bodily changes yet in many instances seeking help and receiving advice did not bring them closer to diagnosis. There are two variants to this typology.

Typology B1: consistent with NICE guidance and no other evidence of system failure (N = 15). This was the most populated typology. Typology B1 captures and expands on the cycles between stages in Walter’s model that could be expected to occur with appropriate management but for presentations that do not fit NICE criteria for a cancer referral. Participants in typology B1 described symptoms that (at least initially) would not have met criteria for an urgent cancer referral or investigation under NICE guidelines [19].

Typology B2: patients describe failure in the pathway or care inconsistent with NICE guidance (N = 11). Typology B2 is closely linked to typology B1, but patients’ accounts include clinical decisions that were clearly inconsistent with application of national guidance or other apparent failures in the diagnostic pathway (e.g. lack of recommended examination such as no rectal exam despite ongoing bowel symptoms, refusal to order diagnostic tests despite clear presence of a red flag symptom, referral letter not being sent).

We group these typologies together not only because they share many common elements but also because it was often hard to decide whether participants’ accounts best fitted B1 or B2. One major reason for this could be the nature of these accounts. Participants may have omitted portions of information, or accentuated negative or positive aspects of an episode in the re-telling, making it difficult to be sure where system failures occurred. This may be an inevitable product of interviewing participants after the cancer is diagnosed, as it can colour their memories of previous clinical encounters. For example, some HCPs were portrayed as heroic, trying to get them help *“I don't know who he overrode*, *but he said "I want a CT scan immediately"*.” By contrast, other HCPs were given the role as villain, denying them access to treatment or admission; *“she was miserable f’ing know it all*, *I’m sorry but it makes my blood boil thinking of her” (Participant 6*, *F*, *head and neck)*.

### Reasons for cycling between appraising and help-seeking

We consider firstly why help-seeking and patient appraisal of bodily changes did not seem to act as a conduit for diagnosis. The following themes apply to both typologies B1 and B2, relating to delays and cyclical factors in the patients’ pathways.

#### HCP appraisal leads patients to re-evaluate their symptoms inappropriately

**Fig 3:**
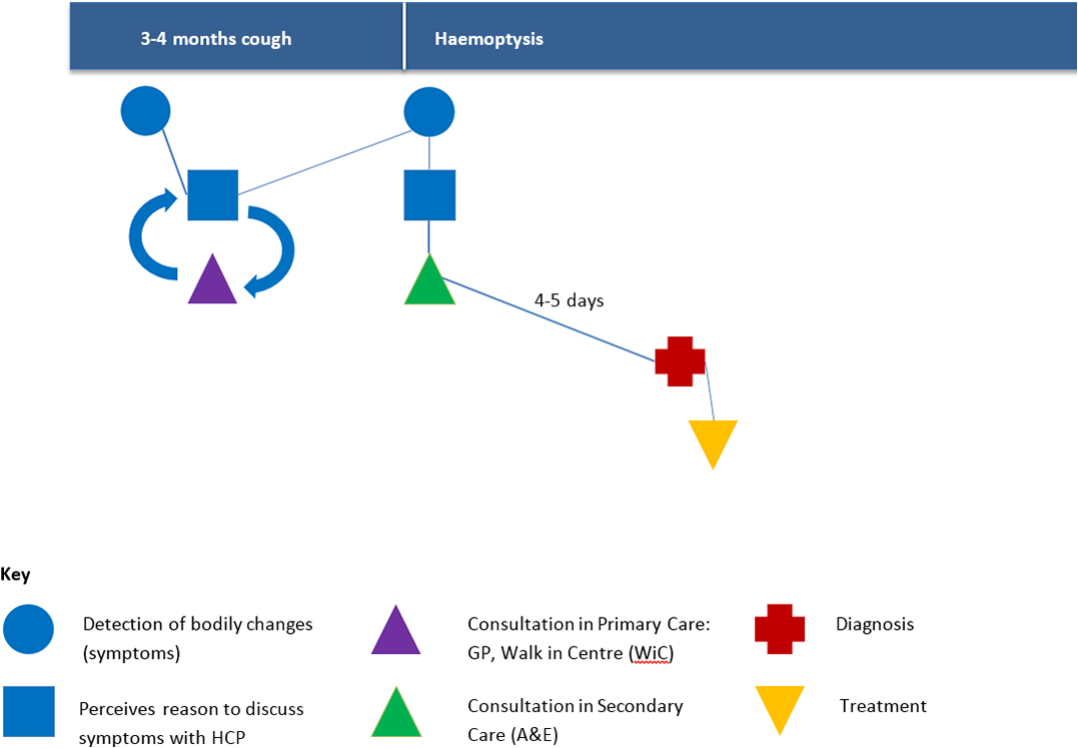
Pathway to treatment illustrating typology B1: Participant 3.

Many participants were given a benign (working) diagnosis, or advised to self-manage symptoms by the GP or ED doctor before their cancer was diagnosed. For example, participant 3 asked his GP repeatedly about symptoms of breathlessness and shoulder pain, which were repeatedly diagnosed as asthma and he was given several types of inhalers (Fig 3):

*To my GP I always said that I felt out of breath but* *he thought because I was asthmatic it had to do with the asthma*. *(Participant 3*, *M*, *lung cancer)*

Many tolerated high levels of pain because they felt it could be explained by what the doctor had said. For example, participant 21 had severe pain in her abdomen. She saw the GP and was prescribed laxatives:

*I couldn’t reach up and get something out of the cupboard*, *I couldn’t peg my washing out*, *I couldn’t hoover*, *I couldn’t do anything like that because it all came back to my tummy and that went on for a while*. *I went to my doctor and she gave me laxatives* *so I thought*, “*okay fair enough*”. *(Participant 21*, *F*, *unknown primary)*

As this quotation suggests, participants felt that the GP’s response was reasonable for what they were experiencing at that stage. Several participants lost confidence in their own appraisal of their symptoms, or rationalised worsening symptoms in light of it. In the extreme, some participants who had particularly long pathways to their cancer diagnosis with lots of contact with HCPs also began to feel badly about their need for continuing help, despite worsening symptoms.

*When I used to go back to them I used to think*, *“Is it me*?*” Because I was depressed I thought that* *maybe I am overreacting sometimes* *and I used to feel guilty going to them to be honest*.*… I was thinking is it really pain or is it in my mind*? *(Participant 2*, *F*, *colorectal)*

#### HCP advice delays help-seeking

Reappraisal of symptoms was linked to help-seeking behaviours in both typology B1 and B2. In one extreme case, a woman who had experienced stomach pains for nearly two months and had been completely constipated for two weeks had begun to vomit faecal matter. She called an ambulance and was subject to blood tests and an x ray in the ED. She was told that “*bad bacteria*” were mixing with the food in her stomach, and that she had gas in her bowel. Her mother convinced her that there was no need to seek further help:

*She said*, *“Well*, *we’ve done everything now*, *…been in by ambulance*, *they’ve seen you*, *they’re saying it’s nothing* *” so we bought the lactulose stuff on the way home*. *(Participant 15*, *F*, *colorectal)*

The participant’s impression from ED doctors that her symptoms amounted to *“nothing”*, rather than being something that might need further attention prevented her from seeking further help from the ED or her regular GP at tis time. It delayed any help-seeking for four days, at which time she bought an enema kit on pharmacist’s advice. Finally, in extreme pain, she sought help again the next day from a GP unit, who sent her to the ED again. She was unconvinced:

*I said “* *What’s the point*? *We’ve been so many times” and [mum] said “well if that’s what they’ve told you to do you’ve got to do it”*. *So we did*. *(Participant 15*, *F*, *colorectal)*

There were several other examples where patients continued to be concerned about their symptoms, but the recent HCP advice to self-manage presented a barrier to returning for more help. They felt they could not ask for further advice or investigations:

*I think to myself* *I cannot go back to the GP*. *The poor GP*, *you know*, *he's tried so I sit this out for a little while*. *(Participant 13*, *F*, *unknown primary)*

*I think if* *I was to live it again*, *I would be more forceful* *and say to them*, *“Look*, *you need to do some tests”*, *because it was the fact that no tests was done for such a long time*, *that maybe it could’ve been sorted out a long time before*. *(Participant 15*, *F*, *colorectal)*

*Perhaps* *it was my fault not to persist* *with going to the GP and asking for further investigation*. *(Participant 14*, *M*, *colorectal)*

### Factors/events enabling patients to reach a diagnosis.

#### Persistent or escalating symptoms

The eventual impetus for investigations leading to a cancer diagnosis often came from a perceived ‘crisis point’ that led patients (or doctors) to appraise symptoms differently: either a further escalation of symptoms, or a decision by the GP that the persistence of symptoms was cause for worry. For example, Participant 27 had been suffering from constipation and abdominal pain for a number of weeks, but approached ED when he started noticing blood:

*I passed blood in my stools […] so that was enough to prompt me to go to A&E because I know it’s dangerous*. *(Participant 27*, *M*, *colorectal)*

However, this was not always successful; sometimes repeated symptoms prompted further primary care visits without resolution. Participant 2 felt that something was wrong with her bowel habits and kept visiting the GP.

*I was complaining and I was going to the doctors constantly and from December I told them*, *“Look I am going to toilet more often*.*” (Participant 2*, *F*, *colorectal)*

Participant 5 had been to the doctor on a number of occasions about blood in her stool, but things had settled down. She went again when her symptoms escalated:

*All of a sudden when I went to go to the toilet to empty my bowels*, *it’s coming out of the front*, *coming out of the vagina*. *So I thought*, *“Don’t know what that’s all about*.*” I’d looked it up*, *funnily enough*, *said it was a fistula*. *I went to the doctors and said*, *“I think I’ve got a fistula*.*” (Participant 5*, *F*, *colorectal)*

#### Circumventing primary care or recommended referral pathways

When the GP pathway had not resulted in resolution, participants sought another way out of the cycle of help-seeking and symptom re-appraisal. In two cases participants described how they bypassed the GP referral pathway deliberately; for example, one participant described going to her regular GP, whom she liked, several times over six months with symptoms that her GP said “*sounds like IBS*”. She then described seeing a different GP on a subsequent occasion, who was *“condescending”* and *“dismissive”*. Just two days later, she decided to go to an emergency department, where her cancer was diagnosed.

There was evidence more widely that patients trusted the ED as a place where tests could be done and thus a conclusive answer would be reached within a short time. Often, they felt this prompt definitive answer would not be or had not been available from primary care. One participant wanted to be *“properly investigated”*:

*Just thought*, *it can’t still just be IBS and I should … even though I don’t really consider it an A&E problem*, *but I probably should go to A&E and have it actually properly investigated*.*[…] I think I just wanted to go somewhere where I would be x-rayed or scanned or just a bit more than 10 minutes with a doctor*. *Something more in depth*. [later in interview] *My doctor will eventually arrange an x-ray or a CT scan or whatever*. *But then they could take months to get a CT scan*, *whereas if you’re there*, *it’s pretty much done*. *(Participant 20*, *F*, *upper GI)*

Patients also recounted examples of GPs and other HCPs using ED as a route to expedite diagnosis. Participants felt validated to approach emergency departments in this way. Over half the sample (17/27) had a direct referral or advice from their GP or another primary care practitioner, e.g. Walk-in-Centre nurse. For example, Participant 16 saw a number of different providers when his symptoms of fever and lack of appetite persisted and the GP’s decision prompted his last ED visit:

*I said okay*, *and from then it was about 8 days*, *I had high temperature*. *I went to the doctors*. *They just said*, *take antibiotics*. *I only went to my GP once though*. *I went to the Emergency more*, *because the GP was usually closed whenever I used to get more ill*. *It used to be a weekend so they don’t open on weekends*. *So I used to go to Ealing Hospital or the Walk-In in Hayes*, *but then when I went to my GP the second time*, *she was*, *you’ve had a high temperature for 8 days so she referred me to A&E in Ealing Hospital*.*(Participant 16*, *M*, *haematological)*

The decision to attend the ED was often portrayed as an instruction, qualified through public messages about inappropriate use of services, specifically the ED: *“I'd phoned NHS Direct so trying not to go to the A&E because they say*, *‘Don’t do that’*.*”*. Some GPs directed a letter or phone call to a named person or hospital service, whilst other HCPs strongly advised participants to attend. For example, one participant was only able to be admitted to hospital if he presented as an emergency, being told there was *“not a thing we can do unless he goes to A&E"*. This was identified as a way to expedite scans and other investigations, and enable swift admission to secondary care. Participants fitting typology A typically received this response at their first visit, whereas those in typology B experienced several visits to a HCP before referral.

However, not all visits to ED led directly to diagnosis. Participant 16 was given antibiotics from the A&E doctor after a cursory examination:

*No*, *they were in a rush to get everyone over and done with*, *because there were a lot of people*. *They were in a rush*. *They actually didn’t listen*. *I go*, *I’ve been having temperatures for a long time*. *They just go*, *oh*, *you need antibiotics*. *[…] As soon as you walked in they go*, *oh*, *what’s the problem*? *You tell them*. *They go*, *listen to your chest*, *look inside your mouth*, *check your temperature*, *and they go*, *oh*, *you just need antibiotics*, *and that’s about it*. *(Participant 16*, *M*, *haematological)*

Despite being x-rayed, participant 4 was sent home with laxatives even after a blockage had been discovered in his bowel. He had to return to ED the same day:

*I think it was the same day or the next day that the vomiting started*, *so I came to hospital*. *They gave me an x-ray*. *They see the blockage there*. *Again they mention constipation*. *I was concerned but in a way relieved that it was constipation*, *so they sent me home with stronger painkillers*. *I started taking those*. *Several hours later I started vomiting and couldn’t hold pills down or water*. *The pain was pretty… up there*. *So I said*, *‘I’ll go to A&E*. *I’m not happy*.*’ My dad took me down there*. *(Participant 4*, *M*, *colorectal)*

### Themes differentiating typologies B2 from B1

#### No referral for NICE-qualifying symptoms

For example, Participant 6 had a persistent ulcer under her tongue. She made repeated appointments with her GP who tried antibiotics, made referrals to dental secondary care, but did not make a two week referral for suspected cancer:

*It wasn’t*, *I’d been up and down to the doctor*, *in the practice*, *I’d been up and down*, *up and down*, *I’ve told her*, *and I’ve told her and tried me on antibiotics*, *no*, *she tried me on painkillers*, *no… and even her she said it could be my teeth*.*(Participant 6*, *F*, *head and neck)*

This symptom (which would fit criteria for referral under the NICE 2005 guidance) also did not trigger a cancer investigation in ED, when the participant was sent there from a walk-in clinic:

*So she [the walk in centre nurse] got through to this department and her actual words were “what do I tell the patient*, *what shall I tell the patient*?*”*, *that was her words and it was like*, *“oh the doctor said if you haven’t heard from us within a week or so then come back”*, *and she gave me some codeine sort of tablets*, *something*, *I can’t re*m*ember the name of it*, *so we came back and I kept looking at… no previously I had… no I didn’t have a copy of that*, *my husband took a copy of the letter*. *(Participant 6*, *F*, *head and neck)*

#### Referral for diagnostic investigation made but investigation did not occur

We found in a number of cases that HCPs made referrals for investigations, but the pathway to diagnosis broke down when these investigations did not go ahead. Participant 8 went to ED for severe abdominal pain. The ED doctor referred her for an ultrasound of her ovaries, but the invitation never arrived and her repeated attempts to get an appointment for ultrasound were unsuccessful. She was in excruciating levels of pain:

*He’s going to write me a referral to see a consultant about the hernia and also have me to have an ultrasound because he thinks it’s something around my ovary and he said to wait*, *we went to the front desk and the nurse at the front desk said there’s no reason to wait and they will send the letter to my house… I think it’s a couple of days one letter came and that one letter was for me to see a consultant about the hernia*, *it was two months in March and the letter for the ultrasound don’t come*, *so since January it was like a roller coaster between my husband going to the hospital*, *arguing because I couldn’t because each day this pain*. *(Participant 8*, *F*, *gynaecological)*

**Fig 4:**
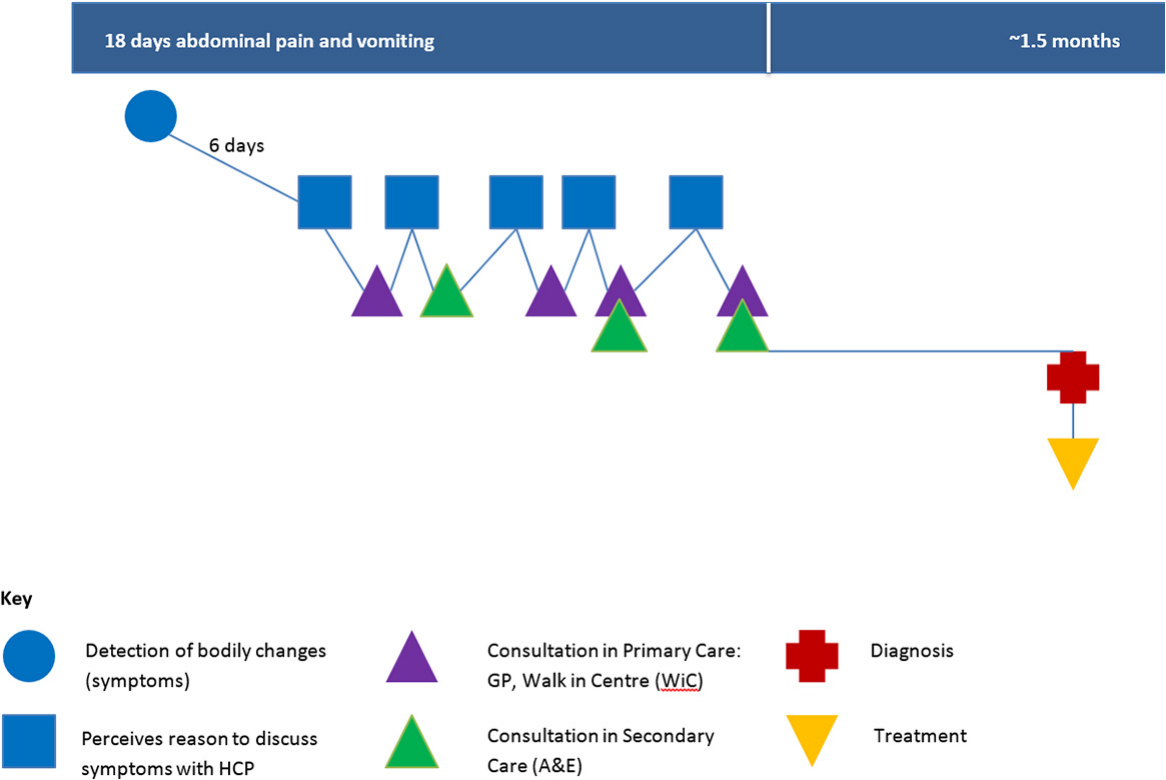
Pathway to treatment illustrating typology B2: Participant 13.

We present Participant 13 as a case study showing a variety of failures in the system (Fig 4). This participant described how her GP sent her to see the surgical registrar at ED due to a palpable abdominal mass, and a history of vomiting for longer than a week. Although the GP had asked for a scan, his request was ignored:

*Eventually*, *Dr*. *Y arrives*, *the surgical registrar*. *He looks at me*, *he says "We'll do a blood test*.*" He says*, *"You look very well*,*" with his sidekick in tow*, *his junior registrar*, *his junior doctor*. *He says*, *"You look very well*.*" He takes some blood tests*, *he has a temporary feel of my tummy*, *he looks at the note of the GP*, *he says*, *"I think we take a minimal approach to this*.*" He says*, *"I think it's gall bladders*, *we'll send you an outpatient appointment and we won't do a scan now*.*" He says to me*, *"Eat bananas*,*" and he sends me home*. *At which point*, *I'm beginning to think that I'm going off my trolley*, *that I'm reacting*. *And I said to him*, *but I'm not fat*, *I'm not 40 and I'm not fertile*. *Gall bladders*, *it's wrong*! *And I don't have that kind of pain*, *you know*. *So he says to me*, *"We'll send you an outpatient appointment*, *and we'll sort this out*.*" (Participant 13*, *F*, *unknown primary)*

Four days later the same participant revisits the ED with another GP referral, this time by telephone. The participant has emergency surgery for bowel obstruction and her tumour is discovered:

*He didn’t phone a colorectal surgeon and say*, *you know*, *just operate–he might have—I doubt it*. *Anyway*, *that night*, *Saturday morning I'm operated on*, *and I'm supposed to be in for short span*, *it's a bowel obstruction*. *Four and a half hours later I'm out*, *and they apparently have removed my whole colon*, *and I have an ileostomy*. *(Participant 13*, *F*, *unknown primary)*

The participant’s surgery was not successful at removing the entire tumour, resulting in the need for further surgery at a different hospital. These participants commonly describe a lack of appreciation by HCPs for the scale of discomfort they experienced, which may affect GP thresholds for referral where CG27 criteria are equivocal. Many participants bore several weeks of severe and debilitating pain, unable to eat with acute bowel and stomach symptoms.

## Discussion

### Main findings

This qualitative study is the first to our knowledge to focus on patients’ experiences of primary and emergency secondary care before a cancer diagnosis. Our identified typologies that represent patients’ experiences of a cancer diagnosis via an emergency pathway suggest most patients identified by this route experienced repeated cycling of appraisal and help-seeking, with or without deviations from an agreed diagnostic process. It identifies sources of delay that are tractable to changes in the healthcare system and thus may be avoidable.

### Strengths and limitations

This study focuses on an under-characterised part of the cancer diagnostic pathway, i.e. amongst patients at particular risk of poor outcomes [3]. Participants were identified prospectively from hospital systems and interviews took place relatively soon (3–6 months) after diagnosis. Thus, most were able to recall their pathway and describe their experiences in rich detail.

We did not focus on one particular cancer, which may limit the extent to which our findings can inform specific cancer pathways. However, other studies suggest there are strong similarities in patient experience of diagnostic delay across different cancer types [14]. Further, the contrasting accounts of pathways to a colorectal cancer diagnosis in our sample illustrate the diversity in patient experience even within one cancer type.

In many instances, it was felt inappropriate by the local clinical team or logistically impossible to invite a patient diagnosed through the emergency route to take part in research. As a result, the number of potential participants approached for interview was limited, with just over 10% of all patients identified as emergency presentations invited. The unpredictable and sometimes intensive nature of urgent care means there are few opportunities to explain a study, invite participation and obtain informed consent whilst patients are in hospital. In addition, once their particular hospital episode has finished, many patients diagnosed with cancer in EDs are very unwell or frail and physically unable to take part in interviews. Studies suggest that emergency presentation for cancer is more likely in older people, particularly over 80 [3, 8]; therefore our sample of participants may have been unrepresentative, with a median age of 59. However, unwell and frail patients took part and wanted to describe their experiences, suggesting clinicians should not automatically consider frailty or ill health insurmountable barriers to inviting similar patients to take part in research.

Patients’ interpretation of what doctors have said may not accord with doctors’ recall or what was recorded in patients’ notes. However, patients’ narratives have “functions other than to provide a strictly accurate and objective history”[20].

### Comparisons with other studies and interpretation of our findings

#### Extending Walter’s model of Pathways to Treatment using typologies

Weller *et al* (2012) recommended using Walter’s model of Pathways to Treatment to standardise reporting of early diagnosis research [14]. In response to their call, “to examine whether these definitions and recommendations can be readily adopted by researchers”, our study provides evidence that it can provide a useful framework for analysis. However, our typologies suggest that prolonged and circuitous pathways are characteristic of this diagnostic route. Whilst these are present in the Walter model, they are not its focus. In agreement with others, we conclude that for an emergency route to diagnosis the model does not represent enough complexity around the causes of delay, the dynamic pathways and the emotional factors at play in help-seeking [21]. Our B1 and B2 typologies highlight diagnostic and interpersonal aspects of clinical encounters that influence symptom appraisal and re-appraisal, leading to delayed help-seeking and scattered approaches to pursuing further clinical advice. Healthcare professionals offering valid symptom management advice may be unaware that their consultation has prevented the patient from returning to visit them. This should be taken into account in models of patient delay. The model could be expanded, therefore, to standardise reporting around advice and symptom management for benign diagnoses, not assuming that malignant appraisals, investigations and tests are necessarily the result of a first help-seeking encounter.

#### Factors driving emergency department attendances

Our finding that over half our sample reported visiting an ED following a GP referral is higher but comparable to the National Cancer Intelligence Network estimate of 30% of cancers diagnosed through emergency admissions as a result of a GP referral [22]. This suggests that when GPs are very concerned about patients’ symptoms, they lack options for rapid investigation under current managed routine or urgent specialist referral routes. Moreover, our study suggests that referring patients to an ED to obtain rapid investigations was not always successful either. Our findings support the need to further improve the transparency and consistency with which primary care can access rapid specialist testing in secondary care when cancer is suspected [23]. This may require more communication between primary and secondary care at the time of referral.

Our study challenges a perception widely reported in the literature that ED use is legitimised when symptoms are at crisis because symptoms were not recognised or ignored until crisis point was reached [24–27]. In contrast, in our study apart from two patients (one who reported ignoring earlier symptoms and another who did not turn up for scheduled investigations), patients did not ignore escalating symptoms and repeatedly sought health care. However, there was no clear pattern of particular symptoms driving urgent care vs. primary care use.

#### Emergency presentation as a marker of avoidable diagnostic delay

Others have discussed the extent to which emergency presentations are markers of *avoidable* delay [7]. In their study of ovarian cancer, Evans *et al* (2007) commented that some delays caused by non-investigation of vague and non-specific cancer symptoms may be unavoidable [28]. Conversely, in this study, participants reported repeated primary care consultations and emergency department visits in response to symptoms that would clearly meet urgent diagnostic referral criteria such as altered bowel habits over weeks or months [17]. The retrospective nature of our interviews may have led participants to place greater importance on certain symptoms than they did at the time. However, it still indicates scope to reduce diagnostic delays through raising awareness of symptoms amongst clinicians in both primary and emergency care services.

Our data also suggest delays may arise because patients lose capacity to appraise worsening symptoms after consulting a healthcare professional. Our results capture the tendency amongst participants in this study to interpret escalating symptoms in light of a presumptive clinical diagnosis, even if tentatively given. This finding is, to some extent, consistent with Walton *et al* (2013) New Zealand study of patients diagnosed with lung cancer following an emergency presentation, which concluded that, “Misplaced patient trust based on long relationships with GPs acted as a barrier to challenging GP opinion” [29].

Whilst trust in, and reluctance to challenge medical opinion were present in our data, there was also a co-construction between doctors and patients that symptoms were not serious. A synthesis of significant event audits indicates that GPs recognise the need to communicate the possibility that the working diagnosis is not the cause of patients’ symptoms [30]. However, patients may adhere more strongly to a benign working diagnosis than GPs intend. Other studies have reported patients delay revisiting the GP because they fear discovering they have cancer [10]. Also, some concerns about the harms or embarrassment of bowel investigations have also been documented [31,32]. These concerns may have acted as a particular incentive for the study’s colorectal cancer patients to adhere strongly to a benign diagnosis. Stronger emphasis on diagnostic uncertainty, therefore, may be needed in situations where the consequences of returning to the GP could be particularly unwelcome.

We suggest that there is a need for greater communication of the ‘safety net’ for patients who meet NICE non-qualifying symptoms for cancer, especially those in severe pain or distress. Lack of safety-netting has been shown to account for some missed lung cancer patients, and a prescriptive rather than promissory approach to safety netting advised [33]. For example, GPs could make the patient a follow up appointment with the advice to cancel if their symptoms improve–in contrast to merely advising to return if they worsen. It has been shown that GPs may give some advice about further help-seeking, but will not offer enough information on how to monitor symptoms or a threshold for re-consultation [31]. Furthermore, it has been advised that patients be told that worsening symptoms will precipitate a referral [34].

#### Experience of diagnosis following an emergency presentation

King *et al* (2011) propose that lack of coordination in the diagnostic process arising from an ED attendance could explain poor outcomes after emergency presentation [11]. Our data indicated instances of poor coordination and potentially avoidable poor experiences amongst participants. Nonetheless, several participants gave glowing accounts of the speed and efficiency of their diagnosis and treatment. Their perceptions of the emergency department may have been particularly positive when compared with prolonged and circuitous experiences of healthcare in the diagnostic pathway up to this point.

## Conclusions

This study captures patients’ experiences across primary and secondary care before a diagnosis of cancer following an emergency presentation, presenting a developed three part typology. It provides novel insights into how these presentations arise, and how patient pathways develop. Most participants needed multiple visits, sometimes to several healthcare providers before visiting ED, and before a cancer diagnosis was made. A minority had a rapid, straightforward pathway. A significant number experienced symptoms on the NICE qualifying list, yet were missed for referral.

Our findings identified potentially avoidable breakdowns in the diagnostic process. In particular, to encourage patients to return to the GP if symptoms escalate, a stronger emphasis is needed on diagnostic uncertainty in discussions between patients and doctors in both primary and secondary care. To improve appropriate access to rapid investigations, systems are needed for primary care to communicate directly with secondary care at the time of referral.

## Supporting Information

S1 Table Research Checklist COREQ Statement. (DOCX)
