# Supplementary material for: Patients’ Experiences of Cancer Diagnosis as a Result of an Emergency Presentation: A Qualitative Study
Source: PLoS One. 2015 Aug 7;10(8):e0135027. doi: 10.1371/journal.pone.0135027 (PMC4529308; doi:10.1371/journal.pone.0135027)
Supplement: S1 Table — (DOCX) [file pone.0135027.s001.docx]

Patients’ experiences of cancer diagnosis as a result of an emergency presentation: a qualitative study

Research Checklist: COREQ Statement

| **No** | **Item** | **Guide questions/description** |  | **Reference** |
| --- | --- | --- | --- | --- |
| **Domain 1: Research team and reflexivity** | | | | |
| **Personal Characteristics** |  |  |  |  |
| 1. | Interviewer/facilitator | Which author/s conducted the interview or focus group? | The authors and other contributors that conducted interviews are listed | Methods, author statements and acknowledgements |
| 2. | Credentials | What were the researcher's credentials? *E.g. PhD, MD* | Interviewers had a minimum of MSc, with two having PhDs. | Title page and acknowledgements |
| 3. | Occupation | What was their occupation at the time of the study? | Interviewers were researchers, a public health registrar and a Manager for Early Diagnosis. | Methods and acknowledgements |
| 4. | Gender | Was the researcher male or female? | All researchers were female | Data not included in the paper to comply with word count guidance but can be added if required |
| 5. | Experience and training | What experience or training did the researcher have? | All researchers collecting data were trained by the lead (GB). | Data not included in the paper to comply with word count guidance but can be added if required |
| **Relationship with participants** |  |  |  |  |
| 6. | Relationship established | Was a relationship established prior to study commencement? | Members of the clinical team first approached patients | Methods |
| 7. | Participant knowledge of the interviewer | What did the participants know about the researcher? e*.g. personal goals, reasons for doing the research* | The reason for doing the research was explained in a participant information sheet and at the start of the interview. | Information sheet available on request |
| 8. | Interviewer characteristics | What characteristics were reported about the interviewer/facilitator? e.g. *Bias, assumptions, reasons and interests in the research topic* | The reason for doing the research was explained in a participant information sheet and at the start of the interview. | Information sheet available on request |
| **Domain 2: study design** | | | | |
| **Theoretical framework** |  |  |  |  |
| 9. | Methodological orientation and Theory | What methodological orientation was stated to underpin the study? *e.g. grounded theory, discourse analysis, ethnography, phenomenology, content analysis* | Thematic analysis | Methods, Analysis |
| **Participant selection** |  |  |  |  |
| 10. | Sampling | How were participants selected? *e.g. purposive, convenience, consecutive, snowball* | Potential participants were identified from a larger service evaluation. The selection was in part purposive (the study sought to maximise the range of patient demographic characteristics) and in part consecutive – all patients that had completed a survey on care and considered well enough to take part were invited to do so. | Methods, Recruitment |
| 11. | Method of approach | How were participants approached? e*.g. face-to-face, telephone, mail, email* | Telephone | Methods, Recruitment |
| 12. | Sample size | How many participants were in the study? | 27 | Results |
| 13. | Non-participation | How many people refused to participate or dropped out? Reasons? | Reasons why participants were not interviewed are listed in the methods. | Methods, Recruitment |
| **Setting** |  |  |  |  |
| 14. | Setting of data collection | Where was the data collected? e*.g. home, clinic, workplace* | Data were collected in hospital or in participants’ homes. | Methods, data collection |
| 15. | Presence of non-participants | Was anyone else present besides the participants and researchers? | Three interviews were conducted with a spouse or relative present, at participants’ request | Methods, data collection |
| 16. | Description of sample | What are the important characteristics of the sample? *e.g. demographic data, date* | Demographics, ethnicity and cancer type are described | Results, table 1. |
| **Data collection** |  |  |  |  |
| 17. | Interview guide | Were questions, prompts, guides provided by the authors? Was it pilot tested? | Guide developed with patient representatives. After the first few interviews, an open-ended structure was used | Methods, data collection |
| 18. | Repeat interviews | Were repeat interviews carried out? If yes, how many? | No | n/a |
| 19. | Audio/visual recording | Did the research use audio or visual recording to collect the data? | Yes | Methods, analysis |
| 20. | Field notes | Were field notes made during and/or after the interview or focus group? | no | Data not included in the paper to comply with word count guidance but can be added if required |
| 21. | Duration | What was the duration of the interviews or focus group? | Interviews ranged in length from ~30 minutes to ~1.5 hours. | Data not included but can be added if required |
| 22. | Data saturation | Was data saturation discussed? | Data saturation was discussed in the analysis as recurrent themes emerged. | Data not included but can be added if required |
| 23. | Transcripts returned | Were transcripts returned to participants for comment and/or correction? | No – participants were often frail and unwell. Their involvement after interviews therefore was limited. Interim findings have been shared at engagement events to elicit feedback | n/a |
| **Domain 3: analysis and findings** | | | | |
| **Data analysis** |  |  |  |  |
| 24. | Number of data coders | How many data coders coded the data? | GB led, JS supplemented the coding | Methods, analysis |
| 25. | Description of the coding tree | Did authors provide a description of the coding tree? | No – the framework for deductive coding is described, relating to Walters Pathways to Treatment | Methods, analysis  A full coding tree can be provided on request |
| 26. | Derivation of themes | Were themes identified in advance or derived from the data? | Themes identified in advance related to Walters Pathway Model. | Methods, analysis |
| 27. | Software | What software, if applicable, was used to manage the data? | NVivo | Methods, analysis |
| 28. | Participant checking | Did participants provide feedback on the findings? | No - participants were often frail and unwell. Their involvement after interviews therefore was limited. Interim findings have been shared at a patient engagement event to elicit general feedback | n/a |
| **Reporting** |  |  |  |  |
| 29. | Quotations presented | Were participant quotations presented to illustrate the themes / findings? Was each quotation identified? e*.g. participant number* | Yes | Results |
| 30. | Data and findings consistent | Was there consistency between the data presented and the findings? | Illustrative quotes or pathways given for all statements, to support themes | Results |
| 31. | Clarity of major themes | Were major themes clearly presented in the findings? | Themes are presented as headings | Results |
| 32. | Clarity of minor themes | Is there a description of diverse cases or discussion of minor themes? | Divergent cases are discussed within each theme | Results |

1. Tong A, Sainsbury P, Craig J (2007) Consolidated criteria for reporting qualitative research (COREQ): a 32-item checklist for interviews and focus groups. *Int J Qual Health Care* 19: 349-357.doi: 10.1093/intqhc/mzm042
